# An *in vitro* study of anti-inflammatory activity of standardised *Andrographis paniculata* extracts and pure andrographolide

**DOI:** 10.1186/s12906-015-0525-7

**Published:** 2015-02-07

**Authors:** Mitchell Low, Cheang S Khoo, Gerald Münch, Suresh Govindaraghavan, Nikolaus J Sucher

**Affiliations:** National Institute of Complementary Medicine, School of Science and Health, University of Western Sydney, Locked Bag 1797, Penrith, N.S.W. 2751, Campbelltown, Australia; Department of Pharmacology and Molecular Medicine Research Group, School of Medicine, University of Western Sydney, Campbelltown, Australia; Network Nutrition-IMCD Australia, Unit 9, 7 Meridian Place, Bella Vista, NSW 2153 Australia; Science Department, Roxbury Community College, 1234 Columbus Avenue, Roxbury Crossing, MA 02120, USA

**Keywords:** Andrographolide, Andrographis paniculata, Anti-inflammatory, Antioxidant, TNF-α, Phytochemistry

## Abstract

**Background:**

The anti-inflammatory activity of *Andrographis paniculata* (Acanthaceae), a traditional medicine widely used in Asia, is commonly attributed to andrographolide, its main secondary metabolite. Commercial *A. paniculata* extracts are standardised to andrographolide content. We undertook the present study to investigate 1) how selective enrichment of andrographolide in commercial *A. paniculata* extracts affects the variability of non-standardised phytochemical components and 2) if variability in the non-standardised components of the extract affects the pharmacological activity of andrographolide itself.

**Methods:**

We characterized 12 commercial, standardised (≥30% andrographolide) batches of *A. paniculata* extracts from India by HPLC profiling. We determined the antioxidant capacity of the extracts using 2,2-diphenyl-1-picrylhydrazyl (DPPH) free radical scavenging, oxygen radical antioxidant capacity (ORAC) and a Folin-Ciocalteu (FC) antioxidant assays. Their anti-inflammatory activity was assessed by assaying their inhibitory effect on the release of tumor necrosis factor alpha (TNF-α) in the human monocytic cell line THP-1.

**Results:**

The andrographolide content in the samples was close to the claimed value (32.2 ± 2.1%, range 27.5 to 35.9%). Twenty-one non-standardised constituents exhibited more than 2-fold variation in HPLC peak intensities in the tested batches. The chlorogenic acid content of the batches varied more than 30-fold. The DPPH free radical scavenging activity varied ~3-fold, the ORAC and FC antioxidant capacity varied ~1.5 fold among batches. In contrast, the TNF-α inhibitory activity of the extracts exhibited little variation and comparison with pure andrographolide indicated that it was mostly due to their andrographolide content.

**Conclusions:**

Standardised *A. paniculata* extracts contained the claimed amount of andrographolide but exhibited considerable phytochemical background variation. DPPH radical scavenging activity of the extracts was mostly due to the flavonoid/phenlycarboxylic acid compounds in the extracts. The inhibitory effect of andrographolide on the release of TNF-α was little affected by the quantitative variation of the non-standardised constituents.

## Background

Chronic inflammation is thought to be a contributing factor to many prevalent ageing-related diseases, such as acute and chronic neurodegenerative diseases, degenerative musculoskeletal diseases, cardiovascular diseases, diabetes, and cancer [1-5]. Other common chronic inflammatory conditions include asthma, rheumatoid arthritis, and inflammatory bowel disease. To date, pharmacotherapy of inflammatory conditions is based mainly on the use of non-steroidal anti-inflammatory drugs (NSAIDs), steroids and more recently tumor necrosis factor alpha (TNF-α) inhibitors [6-8]. However, the prolonged use of NSAIDs can cause serious gastrointestinal toxicity [9]. Some NSAIDs have also been linked to increased blood pressure, greatly increased risk of congestive heart failure and occurrence of thrombosis [10]. These findings illustrate the need to develop novel and safe anti-inflammatory medicines [11].

*Andrographis paniculata* (Acanthaceae), which is endogenous to South India and South East Asia, is used as an herbal medicine in both traditional Indian and Chinese medicine (where it is known as kalmegh and chuanxinlian, respectively) as well as in Malaysia and Thailand [12,13]. *A. paniculata* extracts exhibit anti-inflammatory activity [13] that is commonly attributed to the ent-labdane diterpenoid andrographolide, its characteristic and main secondary metabolite [14-30]. Andrographolide appears to be rapidly absorbed [31] and found to be non-toxic even at very high doses in animals [32] and is well tolerated by humans with no serious adverse effects at doses in the range of 1 to 2 mg/kg/day [33,34]. Intriguingly, andrographolide has been reported to exhibit gastro-protective and ulcer preventive effects, which combined with its well-documented anti-inflammatory effects could make it a safe alternative to traditional NSAIDs [35]. A proprietary *A. paniculata* extract (HMPL-004, Hutchison MediPharma) is under development for the treatment of inflammatory bowel disease [36,37] and is currently being tested in a global phase III clinical trial (http://clinicaltrials.gov/show/NCT01805791).

Commercial *A. paniculata* tablets standardised to requisite concentrations of andrographolide (5% or 30% w/w) are used in clinical studies with an assumption of consistency [38,39]. However with reported innate phytochemical variation influenced by phytogeographical and spatiotemporal factors [40-44], it is not known how selective enrichment (standardization) of andrographolide in commercial preparations affects the variability of non-standardised phytochemical components. It is also not known if variations in the non-standardised components affect the anti-inflammatory activity of the extracts. We undertook the present study to investigate 1) how selective enrichment of andrographolide in commercial *A. paniculata* extracts affects the variability of non-standardised phytochemical components and 2) if variability in the non-standardised components affects the pharmacological activity of the extracts. To this end, we profiled the phytochemical composition and antioxidant capacity of standardised *A. paniculata* extracts and compared the activity of the extracts and purified andrographolide in an assay relevant to their anti-inflammatory activity.

Anti-inflammatory activity of andrographolide has been studied using a number of *in vivo* and *in vitro* experimental paradigms including human whole genome DNA microarrays [20]. The most commonly implicated molecular mechanism underpinning the anti-inflammatory and immunomodulatoy effects of andrographolide is inhibition of the mitogen-activated protein kinase/extracellular signal-regulated kinase (MAPK/ERK) signalling (specifically p38 MAPK/ERK1/2) pathway and downstream transcription factors such as nuclear factor kappa B (NF-κB) and nuclear factor of activated T cells (NFAT) [24,45-50]. An exemplary experimental *in vitro* model where this mechanism has been implicated is the inhibition by andrographolide of the release of TNF-α from LPS stimulated macrophages [22,51-54]. Therefore, we chose TNF-α release from LPS stimulated monocytic leukaemia cells (THP-1) as a model to study anti-inflammatory activity of the extracts and purified andrographolide.

## Methods

### Extract provenance and preparation

Twelve commercial batch samples of *A. paniculata* extracts (extract ratio 14:1) standardised to ≥30% andrographolide were kindly provided to us by LIPA Pharmaceuticals Ltd (NSW, Australia). The extracts conform to the TGA guidelines for incorporation in herbal medicines manufactured in Australia. The whole plant starting material for each batch was sourced (during 2004 – 2008) and the extracts manufactured in India. A systematic botanist authenticated each batch’s starting material and the manufacturer provided traceability documents for each extract. All the samples were re-analysed by high-performance liquid chromatography (HPLC) with photodiode array detection (PDA) for andrographolide content to reconfirm the manufacturer’s certificate of analysis.

### Phytochemical analysis

We used HPLC to profile the phytochemical composition of the *A. paniculata* extracts. Andrographolide (14.3 mg in 10 ml) and extract samples (125 mg in 50 ml) were dissolved by sonication in methanol. HPLC analysis of andrographolide and the extract samples was performed using a Varian Inc. (USA) HPLC system equipped with ProStar 335 photodiode array detector (PDA) and 1200 L quadrupole tandem mass spectrometry (MS/MS) detector. An Alltech Alltima (Alltech Australia) reverse phase C18 column (46 × 150 mm I.D., 5 μm) with a Phenomenex (California, USA) Security C18 guard column (20 mm × 4 mm, 5 μm) were used in these experiments.

We generated HPLC-PDA and HPLC-MS/MS profiles using a 5 μl injection of extract samples. The mobile phase consisted of 0.1% (v/v) aqueous formic acid (mobile phase A) and 0.1% (v/v) formic acid in acetonitrile (mobile phase B). The mobile phase gradient was 10% B for 10 min with a linear increase; to 50% B at 63 min, 70% B at 72 min and then 100% B (wash) for 8 min before equilibrating at the starting composition for 5 min. Mobile phase flow rate was maintained at 1 ml/min. The post column flow was split to send 80% to the PDA (200–500 nm) and 20% to the MS.

The MS conditions were adapted from the work of Dong et al. [55]. Ionization was achieved in positive electrospray ionization mode, scanning between 70–700 m/z with the needle voltage 5000 V at 13 μA; nebulization gas (nitrogen) temperature of 350°C at 20 psi; shield voltage 175 V; capillary voltage 53 V and the detector voltage was 1600 V.

We quantified the andrographolide (0.14 - 1.4 mg/ml) content of the extracts at a detection wavelength of 240 nm and the chlorogenic acid (6.6 – 132.4 μg/ml) content at 330 nm, using five-point linear calibration curves. We generated chromatograms at 227 nm for the detection of diterpenes and at 261 nm and 330 nm for the detection of flavonoids and phenyl carboxylic acids, respectively.

While the PDA was used to quantify andrographolide and chlorogenic acid, the MS detector was used to confirm the identity of these peaks by comparing the MS/MS obtained for the sample and reference standard peaks. MS/MS data was also used to tentatively identify other diterpenes by comparison to published MS data. The UV spectra were used to assign tentatively some of the observed peaks as flavonoids, phenylcarboxylic acids or diterpenes as shown in Table 1.

**Table 1:** **Tentative assignment of HPLC chromatogram peaks as flavonoids, phenylcarboxylic acids or diterpenes based on UV absorbance and MS fragmentation patterns**

**Peakidentitycor number**	**Rt (min)**	**% Area** ^**3**^	**Fold variation** ^**2**^	**UV peaks** ^**1**^ **(nm)**	**MS fragmentation (m/z)** ^**5**^	**Tentative assignment** ^**4**^
Chlorogenic acid	12.2	1.0	34.3	327, 218, 235	Not determined (ND)	Phenyl carboxylic acid
Isoquercetin	28.7	0.9	5.1	204, 255, 353	ND	Flavonol glycoside
Peak 3	29.2	0.6	9.8	323, 219, 234	ND	Phenyl carboxylic acid
Peak 4	29.7	3.5	3.8	325,219,235	ND	Phenyl carboxylic acid
Peak 5	31	1.4	3.4	326, 219, 271	ND	Phenyl carboxylic acid
Peak 6	32.2	4.1	6.1	324, 219, 234	ND	Phenyl carboxylic acid
Peak 7	32.4	3.3	4.5	325, 220	ND	Phenyl carboxylic acid
Peak 8	32.8	3.3	4.7	346, 224, 256	ND	Phenyl carboxylic acid
Peak 9	33.7	2.1	4.5	204, 336, 266	ND	Flavone
Peak 10	36.8	1.3	6.6	327, 219, 235	ND	Phenyl carboxylic acid
Peak 11	37.7	0.8	4.4	327, 271, 223, 204	ND	ND
Andrographolide	38.4	ND	ND	227	351 [M+H]^+^, 333 [M-H_2_O]^+^, 315 [M-2H_2_O]^+^, 297 [M-3H_2_O]^+^	Diterpene
Andropanoside	39.8	1.4	4.8	207	535 [M+K]^+^	Diterpene
Peak 14	40.2	8.0	7.1	225	ND	ND
Peak 15	40.7	4.3	3.7	202	ND	ND
Apigenin	42.2	2.6	6.7	211, 337, 267	ND	Flavone
Wogonoside	45.2	0.7	2.3	265, 212	ND	O-methylated flavone glycoside
Peak18	46.6	0.7	4.6	ND	ND	ND
Peak 19	46.8	0.8	2.5	ND	ND	ND
Neoandrographolide	47.7	9.3	2.7	201	503 [M+Na]^+^,519 [M+K]^+^, 319[M+H-Glu]^+^	Diterpene
Peak 21	48.6	0.5	3.5	ND	ND	ND
Peak 22	49.7	1.5	5.3	ND	ND	ND
Peak 23	50.2	0.76	7.7	ND	ND	ND
Deoxyandrographolide	52.1	6.7	3.7	200	357 [M+Na]^+^, 317 [M+H-H_2_O]^+^, 299 [M+H-2H_2_O]^+^	Diterpene
Dehydroandrographolide	52.6	35.1	2.1	200, 249	355 [M+Na]^+^, 315 [M+H-H_2_O]^+^, 297 [M+H-2H_2_O]^+^	Diterpene
Peak 26	53.6	0.8	2.8	229	ND	ND
Peak 27	54.3	2.0	6.1	229	ND	ND
Peak 28	54.6	2.8	3.0	200, 263	ND	ND

^1^UV peaks are listed in order of intensity.

^2^Fold variation = (Max. peak .area) / (Min. peak area).

^3^Percent (%) area = average peak area from the 12 batches / total combined area × 100. Andrographolide was excluded from the % area calculations.

^4^Assignments based on UV spectrum, MS fragmentation and/or comparison to reference standard RT.

^5^MS fragments are listed in order of intensity.

We used the package ‘msProcess’ [56] (R Project) for Statistical Computing [57] to remove instrumental noise and baseline drift from the chromatograms as described in detail previously [58].

### Antioxidant assays

We performed the DPPH (2,2-di(4-tert-octylphenyl)-1-picrylhydrazyl) radical scavenging capacity, oxygen radical absorbance capacity (ORAC) and the total phenol assay (Folin-Ciocalteu assay; FC) on the extracts as reported in detail previously [58,59].

### Cell culture and tumor necrosis factor α (TNF-α) assay

We cultured human monocytic leukaemia cells (THP-1; American Type Culture Collection, Manassas, VA, USA) in RPMI (Roswell Park Memorial Institute) media containing 4.5 g/l D-Glucose and supplemented with 2 mM GlutaMax, 100 U/ml penicillin, 100 μg/ml streptomycin, and 10% foetal bovine serum at 37°C, 5% CO_2_ in 95% air. Cells (passage number between 10 and 25) were seeded at a density of 1 × 10^5^ cells/well and incubated for 48 h in phorbol-12-myristate-13-acetate (PMA; 100 nM). Non-adherent cells were removed by washing with fresh medium. The remaining cells were pre-incubated with different concentrations of extracts or andrographolide for 1 h and stimulated by lipopolysaccharide (LPS; 50 ng/ml) and interferon gamma (50 units) for 24 h. Andrographolide and extracts were prepared in DMSO and added at a final concentration of 0.1% DMSO. We determined the concentration of TNF-α in the THP-1 culture supernatant by a commercial sandwich ELISA following the manufacturer’s instructions (PeproTech Inc., Rocky Hill, NJ, USA).

Potential cytotoxicity of the extracts and andrographolide was investigated using the MTT (3-(4,5-dimethylthiazol-2-yl)-2,5-diphenyltetrazolium bromide) assay. No toxic effects of the extracts or andrographolide were observed at the tested concentrations (data not shown).

### Statistical data analysis

All data is reported as mean ± standard deviation of the average of three replicates in experiments performed on three separate days. The TNF-α dose response data were fitted with a log (inhibitor) vs. normalized response with variable slope model using Prism 5 for Mac OSX (GraphPad Software, La Jolla, CA). IC_50_ values were calculated from the fitted curves. F-test was used to compare if the best-fit values differed between the data sets. Differences were considered statistically significant if *p* < 0.05.

## Results and discussion

**Figure 1:**
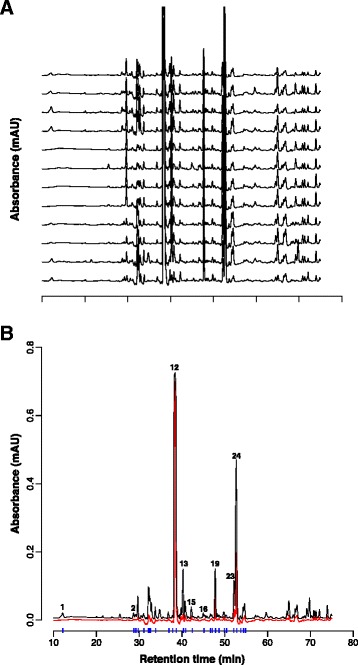
**Chromatograms of standardised** ***A. paniculata*** **extracts. A)** The chromatograms of twelve batches (#1 at the bottom, #12 at the top) standardised to contain 30% (w/w) andrographolide were recorded at 227 nm. **B)** Composite chromatograms illustrating the variation between the HPLC profiles of the 12 batches. The black chromatogram was generated using the highest peaks from each of the 12 extracts. The red chromatogram was generated using the smallest peaks. Vertical blue lines indicate the retention time of the peaks that were used for the quantitative analysis of batch-to-batch variation (see Table 1). Numbered peaks were identified as chlorogenic acid (#1), isoquercetin (#2), andrographolide (#12), andropanoside (#13), apigenin (#15), wogonoside (#16), neoandrogrpapholide (#19), deoxyandrographolide (#23), and 14-deoxy-11,12-didehydroandrographolide (#24).

The phytochemical composition of 12 *A.paniculata* extracts was characterized by HPLC-PDA (Figure 1A). Consistent with the intended standardization, to contain 30% andrographolide, the content was on average close to this value (32.2 ± 2.1%, range 27.5 to 35.9%; *n* = 12; peak #12 in Figure 1B). Correlation analysis of the andrographolide concentration versus storage time revealed that there was no observable loss in andrographolide concentration due to storage of the dry extracts (r^2^ = 0.03; not illustrated). Non-standardised constituents other than diterpenes (e. g. peaks 1–11), varied to a greater degree between the batches (Table 1). For example, the chlorogenic acid content exhibited >30 fold variation between batches (0.1 to 3.5 mg per g of extract; mean 1.4 ± 1.1 mg/g). The maximum variation observed between the chromatograms is illustrated by the red curve in Figure 1B.

**Figure 2:**
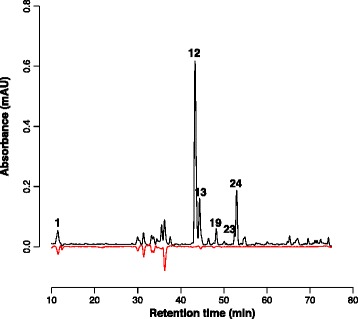
**Online DPPH assay.** Representative chromatograms of *A. paniculata* extract #9. The chromatogram at 227 nm (black line) is contrasted with the DPPH absorbance at 529 nm (red line). Compounds that have DPPH antioxidant activity are observed as a negative peak at 529 nm. Numbered peaks were identified as chlorogenic acid (#1), andrographolide (#12), andropanoside (#13), neoandrogrpapholide (#19), deoxyandrographolide (#23), and 14-deoxy-11,12-didehydroandrographolide (#24).

The phytochemical analysis of the extracts revealed considerable variability in the peak intensities of the non-standardised constituents, some of which belonged to the flavonoid and phenylcarboxylic acid class. Flavonoids and phenolic acids are known to be good free radical scavengers and antioxidants [60]. Oxidative stress is believed to contribute to inflammatory tissue damage and play a role cytokine signalling [61]. We therefore wondered to what degree the observed phytochemical variation might be reflected in the antioxidant capacity of the extracts [62]. We used three non-cellular assays to measure the DPPH radical scavenging capacity, oxygen radical absorbance capacity (ORAC) and total phenol content (FC assay) [58]. The average DPPH radical scavenging capacity of the total extracts was 81.6 ± 30.9 μmol/g gallic acid equivalent and varied 3 fold. DPPH reactivity was correlated with the variation in chlorogenic acid content (r^2^ = 0.8). Online-HPLC revealed that the DPPH scavenging was mainly due to peaks 1–11 (flavonoids and phenylcarboxylic acids) while the diterpenes (peaks #12, 13, 20, 24 and 25) were virtually devoid of DPPH radical scavenging capacity under the conditions of our experiments (Figure 2). The average ORAC of the extracts was 1.05 ± 0.16 mmol/g gallic acid equivalent and varied 1.6 fold. The average antioxidant activity of the extracts in the FC assay was 0.40 ± 0.05 mmol/g gallic acid equivalent and varied 1.5 fold.

**Figure 3:**
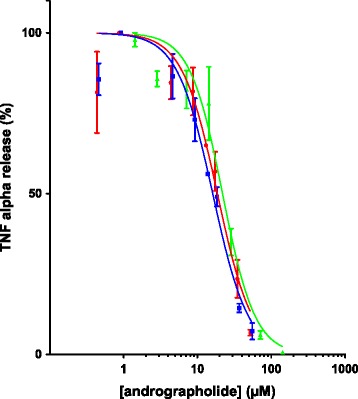
**Dose response curves for batches #9 (blue, squares), #3 (red, circles) and andrographolide (green, triangles).** IC_50_ of pure andrographolide: 21.9 μM (*n* = 3; 95% confidence interval: 18.1 - 26.5 μM), extract #3: 16.4 μM (*n* = 3; 95% confidence interval: 13.8 - 18.8 μM), extract #9: 18.7 μM (*n* = 3; 95% confidence interval: 14.9 - 23.4 μM). The concentrations have been normalized to concentration of andrographolide in the batches. The data were fitted with a log (inhibitor) vs. normalized response with variable slope model. The IC_50_ was calculated from the fitted curve. F-test indicated that all data could be equally fitted with a single curve with IC_50_ = 18.5 μM (*p* = 0.51; 95% confidence interval: 16.7 to 20.7 μM).

Next, we assayed the activity of the extracts and purified andrographolide on the inhibition of TNF-α release by LPS stimulated THP-1 cells. For this experiment, we obtained dose response curves of purified andrographolide and the extracts #3 and #9, the extracts with highest and lowest DPPH free radical scavenging activity. The results revealed that the dose response curves obtained upon application of pure andrographolide or the extracts were very similar (Figure 3), when normalized to andrographolide concentration. The half maximal inhibitory concentration (IC_50_) of pure andrographolide was 21.9 μM (*n* = 3; 95% confidence interval: 18.1 - 26.5 μM) compared to 16.4 μM (*n* = 3; 95% confidence interval: 13.8 - 18.8 μM) and 18.7 μM (*n* = 3; 95% confidence interval: 14.9 - 23.4 μM) for extracts #3 and #9, respectively. Thus, the phytochemical background variation of the extracts appeared not to influence significantly the activity of andrographolide in this *in vitro* assay.

We characterized the phytochemical composition of 12 batches of commercial *A. paniculata* extracts standardised for andrographolide content (30% w/w). We confirmed that the extracts contained the specified amount of andrographolide but observed substantial variation in the non-standardised components of the extracts. Chlorogenic acid exhibited maximal (~30 fold) variation. This acid is one of the most abundant phenolic compounds in the human diet and is present in significant amounts in coffee [63]. Various pharmacological effects of chlorogenic acid have been described and recent interest has focused on its effects on glucose and lipid metabolism [63]. Hypoglycaemic and hypocholesterolemic effects of water and ethanol *A. paniculata* extracts have been reported but the role of chlorogenic acid and its other phytochemical constituents and the molecular mechanisms underpinning these effects remain to be established [64]. Patients with diabetes taking *A. paniculata* extracts may therefore need extra monitoring and dietary counselling upon commencing or changing *A. paniculata* extract containing medications due to batch-to-batch variability of chlorogenic acid and its potential effects on blood glucose levels.

Our results revealed that of the commonly used *in vitro* antioxidant assays the DPPH assay was a more sensitive indicator of the phytochemical background variation in the standardised extracts than the ORAC and FC assays. The absolute increase in the activity of the extracts and reduced inter-extract variation observed in the ORAC and FC assays may be due to the activity of andrographolide and other diterpenes (and possibly additional unidentified compounds), which did not exhibit activity in the DPPH assay. The results suggest that at least under the conditions of our experiments the diterpenes mainly functioned via hydrogen atom transfer (HAT) but not electron transfer (ET) in contrast to the flavonoids and phenyl carboxylic acids which exhibited activity in both the HAT and ET mechanisms [58]. The DPPH reactivity was correlated with the variation in chlorogenic acid (r^2^ = 0.8), illustrating that chlorogenic acid contributed significantly to the ET antioxidant activity of the extracts. The chlorogenic acid quantity showed no correlation with the ORAC or FC results, despite chlorogenic acid being a HAT antioxidant; this is likely due to andrographolide masking chlorogenic acids contribution to the total HAT activity as andrographolide is in much greater abundance (~100 times).

There are many anti-inflammatory compounds reported in *A. paniculata* [52,65,66] but andrographolide is the most abundant [55]. In this study the most dissimilar extracts in terms of DPPH activity and chemical profile (#3 and #9) were compared to pure andrographolide to assess their inhibition of TNF α release from LPS stimulated macrophages. Our comparison between the purified andrographolide and extracts containing parallel amounts of andrographolide in this assay found very similar dose response curves. Thus, at this level of analysis, there was no evidence for antagonistic, additive or synergistic effects between andrographolide and other phytochemical constituents of the extracts. The extract activity was almost entirely accounted for by the andrographolide content, thus the contribution of the other anti-inflammatory compounds present was minor, likely due to their lower abundance.

The IC_50_ of andrographolide (21.9 μM) was comparable to values obtained in a similar assay using mouse peritoneal macrophages [51]. A number of additional *in vitro* studies using various experimental paradigms have all reported effective concentrations of pure andrographolide in the range of 7 to 35 μM [20,24,45,47,48,66-68]. The concentration reported in ours and other *in vitro* studies are nominal concentrations and the “actual” concentration at the site of interaction between andrographolide and its potential (extra- and/or intracellular) target proteins (receptor(s) or enzymes) has not been determined. Nonetheless, it is noteworthy that the IC_50_ of andrographolide in our *in vitro* assay was ~50 times higher than the maximal plasma concentration (0.5 μM) achieved following oral administration of 50 mg andrographolide in healthy human volunteers [69], although significantly higher steady state blood concentrations (1.9 μM) have been reported in humans taking ~1 mg andrographolide per kg body weight per day [31]. In a prospective clinical study for the relief of rheumatoid arthritis symptoms, Burgos and colleagues administered 3 times per day 100 mg of *A. paniculata* extract standardised to 30% andrographolide [38]. Although these authors observed some positive effects, the *in vitro* data suggest that the administered dose might have been at the low end of the effective dose range and future clinical studies should consider testing higher doses. It will be interesting to investigate whether or not the absorption, distribution, metabolism and excretion of andrographolide alone or andrographolide administered as *A. paniculata* extract (with variable phytochemical background) differ or not.

## Conclusion

Supported by a considerable body of published evidence, standardisation of *A. paniculata* extracts for andrographolide content is based on the notion that this compound accounts for the pharmacological effects of the complex extracts. To the best of our knowledge, however, our data represent the only quantitative direct comparison of the efficacy of pure andrographolide and *A. paniculata* extracts. Thus, our results support the development of andrographolide or andrographolide-derived compounds as anti-inflammatory drugs. Interestingly, *A. paniculata* related drug development efforts presently include both herbal medicine based approaches in the form of standardised complex extracts as well as orthodox, synthetic drug based approaches [40,70-79]. We believe that well characterized standardised *A. paniculata* extracts present an excellent opportunity to further investigate the advantages and disadvantages of the herbal vs. synthetic approach in the treatment of inflammatory conditions.

## References

[1] Wyss-Coray T (2006). Inflammation in Alzheimer disease: driving force, bystander or beneficial response?. Nat Med.

[2] Hansson GK (2005). Inflammation, atherosclerosis, and coronary artery disease. N Engl J Med.

[3] Balkwill F, Charles KA, Mantovani A (2005). Smoldering and polarized inflammation in the initiation and promotion of malignant disease. Cancer Cell.

[4] Wellen KE, Hotamisligil GS (2005). Inflammation, stress, and diabetes. J Clin Invest.

[5] Tabas I, Glass CK (2013). Anti-inflammatory therapy in chronic disease: challenges and opportunities. Science.

[6] Brooks PM, Day RO (1991). Nonsteroidal antiinflammatory drugs–differences and similarities. N Engl J Med.

[7] St Clair EW (2002). Tides of inflammation: impact of biologics. J Rheumatol Suppl.

[8] Canvin JM, El-Gabalawy HS (1999). Anti-inflammatory therapy. Phys Med Rehabil Clin N Am.

[9] Wolfe MM, Lichtenstein DR, Singh G (1999). Gastrointestinal toxicity of nonsteroidal antiinflammatory drugs. N Engl J Med.

[10] Pirmohamed M, James S, Meakin S, Green C, Scott AK, Walley TJ (2004). Adverse drug reactions as cause of admission to hospital: prospective analysis of 18 820 patients. BMJ.

[11] McCarberg B, Gibofsky A (2012). Need to develop new nonsteroidal anti-inflammatory drug formulations. Clin Ther.

[12] Chao W-W, Lin B-F (2010). Isolation and identification of bioactive compounds in Andrographis paniculata (Chuanxinlian). Chin Med.

[13] Akbar S (2011). Andrographis paniculata: a review of pharmacological activities and clinical effects. Altern Med Rev: J Clin Ther.

[14] Cava MP, Chan WR, Haynes LJ, Johnson LF, Weinstein B (1962). The structure of andrographolide. Tetrahedron.

[15] Jarukamjorn K, Nemoto N (2008). Pharmacological aspects of Andrographis paniculata on health and its major diterpenoid constituent andrographolide. J Health Sci.

[16] Xia YF, Ye BQ, Li YD, Wang JG, He XJ, Lin X (2004). Andrographolide attenuates inflammation by inhibition of NF-kappa B activation through covalent modification of reduced cysteine 62 of p50. J Immunol.

[17] Chen HW, Lin AH, Chu HC, Li CC, Tsai CW, Chao CY (2011). Inhibition of TNF-alpha-Induced Inflammation by andrographolide via down-regulation of the PI3K/Akt signalling pathway. J Nat Prod.

[18] Chandrasekaran CV, Murali B, Deepak M, Agarwal A (2012). *In vitro* comparative evaluation of non-leaves and leaves extracts of Andrographis paniculata on modulation of inflammatory mediators. Anti-Inflamm Anti-Allergy Agents Med Chem.

[19] Chandrasekaran CV, Thiyagarajan P, Deepak HB, Agarwal A (2011). *In vitro* modulation of LPS/calcimycin induced inflammatory and allergic mediators by pure compounds of Andrographis paniculata (King of bitters) extract. Int Immunopharmacol.

[20] Parichatikanond W, Suthisisang C, Dhepakson P, Herunsalee A (2010). Study of anti-inflammatory activities of the pure compounds from Andrographis paniculata (burm.f.) Nees and their effects on gene expression. Int Immunopharmacol.

[21] Chandrasekaran CV, Gupta A, Agarwal A (2010). Effect of an extract of Andrographis paniculata leaves on inflammatory and allergic mediators *in vitro*. J Ethnopharmacol.

[22] Chao WW, Kuo YH, Lin BF (2010). Anti-inflammatory activity of new compounds from Andrographis paniculata by NF-kappaB transactivation inhibition. J Agric Food Chem.

[23] Sheeja K, Shihab PK, Kuttan G (2006). Antioxidant and anti-inflammatory activities of the plant Andrographis paniculata Nees. Immunopharmacol Immunotoxicol.

[24] Shen T, Yang WS, Yi YS, Sung GH, Rhee MH, Poo H (2013). AP-1/IRF-3 Targeted Anti-Inflammatory Activity of Andrographolide Isolated from Andrographis paniculata. Evidence-Based Complementary Altern Med.

[25] Shen YC, Chen CF, Chiou WF (2000). Suppression of rat neutrophil reactive oxygen species production and adhesion by the diterpenoid lactone andrographolide. Planta Med.

[26] Shen YC, Chen CF, Chiou WF (2002). Andrographolide prevents oxygen radical production by human neutrophils: possible mechanism(s) involved in its anti-inflammatory effect. Br J Pharmacol.

[27] Jayakumar T, Hsieh C-Y, Lee J-J, Sheu J-R (2013). Experimental and Clinical Pharmacology of Andrographis paniculata and Its Major Bioactive Phytoconstituent Andrographolide. Evidence-Based ComplementaryAltern Med.

[28] Abu-Ghefreh AA, Canatan H, Ezeamuzie CI (2009). *In vitro* and *in vivo* anti-inflammatory effects of andrographolide. Int Immunopharmacol.

[29] Levita J, Nawawi A, Mutholib A, Ibrahim S (2010). Andrographolide inhibits COX-2 expression in human fibroblast cells Due to its interaction with arginine and histidine in cyclooxygenase site. J Appl Sci.

[30] Levita J, Nawawi A, Mutholib A, Ibrahim S (2010). Andrographolide: a review of its anti-inflammatory activity via inhibition of NF-kappaB activation from computational chemistry aspects. Int J Pharmacol.

[31] Panossian A, Hovhannisyan A, Mamikonyan G, Abrahamian H, Hambardzumyan E, Gabrielian E (2000). Pharmacokinetic and oral bioavailability of andrographolide from Andrographis paniculata fixed combination Kan Jang in rats and human. Phytomedicine.

[32] Sithisomwongse N, Phengchata J, Cheewapatana S (1989). Acute and chronic toxicity of Andrographis paniculata Nee. Th Thai J Pharm Sci.

[33] Gabrielian ES, Shukarian AK, Goukasova GI, Chandanian GL, Panossian AG, Wikman G (2002). A double blind, placebo-controlled study of Andrographis paniculata fixed combination Kan Jang in the treatment of acute upper respiratory tract infections including sinusitis. Phytomedicine.

[34] Melchior J, Spasov AA, Ostrovskij OV, Bulanov AE, Wikman G (2000). Double-blind, placebo-controlled pilot and phase III study of activity of standardised Andrographis paniculata Herba Nees extract fixed combination (Kan jang) in the treatment of uncomplicated upper-respiratory tract infection. Phytomedicine.

[35] Saranya P, Geetha A, Selvamathy SM (2011). A biochemical study on the gastroprotective effect of andrographolide in rats induced with gastric ulcer. Indian J Pharm Sci.

[36] Sandborn WJ, Targan SR, Byers VS, Rutty DA, Mu H, Zhang X (2013). Andrographis paniculata extract (HMPL-004) for active ulcerative colitis. Am J Gastroenterol.

[37] Michelsen KS, Wong MH, Ko B, Thomas LS, Dhall D, Targan SR (2013). HMPL-004 (Andrographis paniculata extract) prevents development of murine colitis by inhibiting T-cell proliferation and TH1/TH17 responses. Inflamm Bowel Dis.

[38] Burgos RA, Hancke JL, Bertoglio JC, Aguirre V, Arriagada S, Calvo M (2009). Efficacy of an Andrographis paniculata composition for the relief of rheumatoid arthritis symptoms: a prospective randomized placebo-controlled trial. Clin Rheumatol.

[39] Caceres DD, Hancke JL, Burgos RA, Sandberg F, Wikman GK (1999). Use of visual analogue scale measurements (VAS) to asses the effectiveness of standardised Andrographis paniculata extract SHA-10 in reducing the symptoms of common cold. A randomized double blind-placebo study. Phytomedicine.

[40] Chen JX, Xue HJ, Ye WC, Fang BH, Liu YH, Yuan SH (2009). Activity of andrographolide and its derivatives against influenza virus *in vivo* and *in vitro*. Biol Pharm Bull.

[41] Bhan MK, Dhar AK, Khan S, Lattoo SK, Gupta KK, Choudhary DK (2006). Screening and optimization of Andrographis paniculata (Burm.f.) Nees for total andrographolide content, yield and its components. Sci Hortic.

[42] Pholphana N, Rangkadilok N, Saehun J, Ritruechai S, Satayavivad J (2013). Changes in the contents of four active diterpenoids at different growth stages in Andrographis paniculata (Burm.f.) Nees (Chuanxinlian). Chin Med.

[43] Saravanan R, Krishti S, Gajbhiye NA, Maiti S (2008). Influence of light intensity on gas exchange, herbage yield and andrographolide content in Andrographis paniculata (Nees.). Indian J Horticulture.

[44] Patarapanich C, Laungcholatan S, Mahaverawat N, Chaichantipayuth C, Pummangura S (2007). HPLC determination of active diterpene lactones from Andrographis paniculata Nees planted in various seasons and regions in Thailand. Thai J Pharm Sci.

[45] Tsai H-R, Yang L-M, Tsai W-J, Chiou W-F (2004). Andrographolide acts through inhibition of ERK1/2 and Akt phosphorylation to suppress chemotactic migration. Eur J Pharmacol.

[46] Carretta MD, Alarcon P, Jara E, Solis L, Hancke JL, Concha II (2009). Andrographolide reduces IL-2 production in T-cells by interfering with NFAT and MAPK activation. Eur J Pharmacol.

[47] Lu WJ, Lin KH, Hsu MJ, Chou DS, Hsiao G, Sheu JR (2012). Suppression of NF-kappaB signalling by andrographolide with a novel mechanism in human platelets: regulatory roles of the p38 MAPK-hydroxyl radical-ERK2 cascade. Biochem Pharmacol.

[48] Lee WR, Chung CL, Hsiao CJ, Chou YC, Hsueh PJ, Yang PC (2012). Suppression of matrix metalloproteinase-9 expression by andrographolide in human monocytic THP-1 cells via inhibition of NF-kappaB activation. Phytomedicine.

[49] de Las Heras B, Hortelano S (2009). Molecular basis of the anti-inflammatory effects of terpenoids. Inflamm Allergy Drug Targets.

[50] Wang W, Wang J, Dong SF, Liu CH, Italiani P, Sun SH (2010). Immunomodulatory activity of andrographolide on macrophage activation and specific antibody response. Acta Pharmacol Sin.

[51] Zhi-wu Z, Lin-hua Q (2011). Andrographolide inhibits expression of TNF-α and IL-12 in activated macrophages. Acad JSecond Mil Med Univ.

[52] Liu J, Wang ZT, Ge BX (2008). Andrograpanin, isolated from Andrographis paniculata, exhibits anti-inflammatory property in lipopolysaccharide-induced macrophage cells through down-regulating the p38 MAPKs signalling pathways. Int Immunopharmacol.

[53] Ravipati AS, Zhang L, Koyyalamudi SR, Jeong SC, Reddy N, Bartlett J (2012). Antioxidant and anti-inflammatory activities of selected Chinese medicinal plants and their relation with antioxidant content. BMC Complement Altern Med.

[54] Chao WW, Kuo YH, Hsieh SL, Lin BF. Inhibitory effects of ethyl acetate extract of Andrographis paniculata on NF-{kappa}B trans-activation activity and LPS-induced acute inflammation in mice. Evidence-Based Complementary and Alternative Medicine, vol. 2011, Article ID 254531, 9 pages, 2011. doi:10.1093/ecam/nep120.

[55] Dong HJ, Zhang ZJ, Yu J, Liu Y, Xu FG (2009). Chemical fingerprinting of Andrographis paniculata (Burm. f.) Nees by HPLC and hierarchical clustering analysis. J Chromatogr Sci.

[56] Gong L, Constantine W, Chen YA. Protein Mass Spectra Processing. In: Protein Mass Spectra Processing. TIBCO Software Inc.; 2007–2009.

[57] Team RCD (2010). A Language Environment for Statistical Computing. A Language Environment for Statistical Computing.

[58] Cook R, Hennell JR, Lee S, Khoo CS, Carles MC, Higgins VJ (2013). The Saccharomyces cerevisiae transcriptome as a mirror of phytochemical variation in complex extracts of Equisetum arvense from America, China, Europe and India. BMC Genomics.

[59] Steele ML, Truong J, Govindaraghavan S, Ooi L, Sucher NJ, Munch G (2013). Cytoprotective properties of traditional Chinese medicinal herbal extracts in hydrogen peroxide challenged human U373 astroglia cells. Neurochem Int.

[60] Rice-Evans CA, Miller NJ, Paganga G (1996). Structure-antioxidant activity relationships of flavonoids and phenolic acids. Free Radic Biol Med.

[61] Kim JY, Ro JY (2005). Signal pathway of cytokines produced by reactive oxygen species generated from phorbol myristate acetate-stimulated HMC-1 cells. Scand J Immunol.

[62] Huang D, Ou B, Prior RL (2005). The chemistry behind antioxidant capacity assays. J Agric Food Chem.

[63] Meng S, Cao J, Feng Q, Peng J, Hu Y (2013). Roles of chlorogenic acid on regulating glucose and lipids metabolism: a review. Evidence-based Complementary Altern Med.

[64] Kumar A, Dora J, Singh A, Tripathi R (2012). A review on king of bitter (Kalmegh). Int J Res Pharm Chem.

[65] Zhao J, Yang G, Liu H, Wang D, Song X, Chen Y (2002). Determination of andrographolide, deoxyandrographolide and neoandrographolide in the Chinese herb Andrographis paniculata by micellar electrokinetic capillary chromatography. Phytochem Anal.

[66] Batkhuu J, Hattori K, Takano F, Fushiya S, Oshiman K, Fujimiya Y (2002). Suppression of NO production in activated macrophages *in vitro* and *ex vivo* by neoandrographolide isolated from Andrographis paniculata. Biol Pharm Bull.

[67] Chiou WF, Lin JJ, Chen CF (1998). Andrographolide suppresses the expression of inducible nitric oxide synthase in macrophage and restores the vasoconstriction in rat aorta treated with lipopolysaccharide. Br J Pharmacol.

[68] Chiou WF, Chen CF, Lin JJ (2000). Mechanisms of suppression of inducible nitric oxide synthase (iNOS) expression in RAW 264.7 cells by andrographolide. Br J Pharmacol.

[69] Song Y-X, Liu S-P, Jin Z, Qin J-F, Jiang Z-Y (2013). Qualitative and quantitative analysis of Andrographis paniculata by rapid resolution liquid chromatography/time-of-flight mass spectrometry. Molecules.

[70] Hai-Wei X, Gui-Fu D, Gai-Zhi L, Jun-Feng W, Hong-Min L (2007). Synthesis of andrographolide derivatives: a new family of alpha-glucosidase inhibitors. Bioorg Med Chem.

[71] Jada SR, Matthews C, Saad MS, Hamzah AS, Lajis NH, Stevens MF (2008). Benzylidene derivatives of andrographolide inhibit growth of breast and colon cancer cells *in vitro* by inducing G(1) arrest and apoptosis. Br J Pharmacol.

[72] Li J, Huang W, Zhang H, Wang X, Zhou H (2007). Synthesis of andrographolide derivatives and their TNF-alpha and IL-6 expression inhibitory activities. Bioorg Med Chem Lett.

[73] Menon V, Bhat S (2010). Anticancer activity of andrographolide semisynthetic derivatives. Nat Prod Commun.

[74] Suebsasana S, Pongnaratorn P, Sattayasai J, Arkaravichien T, Tiamkao S, Aromdee C (2009). Analgesic, antipyretic, anti-inflammatory and toxic effects of andrographolide derivatives in experimental animals. Arch Pharm Res.

[75] Wang B, Li J, Huang WL, Bin Zhang H, Qian H, Zheng YT (2011). Synthesis and biological evaluation of andrographolide derivatives as potent anti-HIV agents. Chin Chem Lett.

[76] Wang Z, Yu P, Zhang G, Xu L, Wang D, Wang L (2010). Design, synthesis and antibacterial activity of novel andrographolide derivatives. Bioorg Med Chem.

[77] Xu H-W, Dai G-F, Liu G-Z, Wang J-F, Liu H-M (2007). Synthesis of andrographolide derivatives: a new family of α-glucosidase inhibitors. Bioorg Med Chem.

[78] Xu HW, Zhang JY, Liu HM, Wang JF (2006). Synthesis of andrographolide cyclophosphate derivatives and their antitumor activities. Synth Commun.

[79] Zhang B, Yan L, Zhou P, Dong Z, Feng S, Liu K (2013). CHP1002, a novel andrographolide derivative, inhibits pro-inflammatory inducible nitric oxide synthase and cyclooxygenase-2 expressions in RAW264.7 macrophages via up-regulation of heme oxygenase-1 expression. Int Immunopharmacol.

